# A qualitative study to explore daily versus on-demand oral pre-exposure prophylaxis (PrEP) in young people from South Africa, Uganda and Zimbabwe

**DOI:** 10.1371/journal.pone.0287627

**Published:** 2023-06-29

**Authors:** Janan Janine Dietrich, Nadia Ahmed, Gugulethu Tshabalala, Minju Wu, Mamakiri Mulaudzi, Stefanie Hornschuh, Millicent Atujuna, Richard Muhumuza, Andrew Sentoogo Ssemata, Lynda Stranix-Chibanda, Teacler Nematadzira, Linda-Gail Bekker, Neil Martinson, Janet Seeley, Julie Fox

**Affiliations:** 1 Perinatal HIV Research Unit, Faculty of Health Sciences, University of the Witwatersrand, Johannesburg, South Africa; 2 Health Systems Research Unit, South African Medical Research Council, Bellville, South Africa; 3 African Social Sciences Unit of Research and Evaluation (ASSURE), Faculty of Health Sciences, University of the Witwatersrand, Johannesburg, South Africa; 4 Mortimer Market Centre, Central North West London NHS Trust, London, United Kingdom; 5 Desmond Tutu HIV Centre, University of Cape Town, Cape Town, South Africa; 6 Harvard College, Cambridge, MA, United States of America; 7 Medical Research Council/Uganda Virus Research Institute and London School of Hygiene & Tropical Medicine Uganda Research Unit, Entebbe, Uganda; 8 Department of Global Health and Development, London School of Hygiene and Tropical Medicine, London, United Kingdom; 9 University of Zimbabwe Clinical Trials Research Centre, Faculty of Medicine and Health Sciences, Harare, Zimbabwe; 10 King’s College London, London, United Kingdom; Oregon State University, UNITED STATES

## Abstract

**Background:**

Adolescents in sub-Saharan Africa (SSA) remain vulnerable to HIV infection. While pre-exposure prophylaxis (PrEP) is highly effective in preventing HIV transmission as a daily or on-demand regimen, tailored approaches are necessary. The Combined HIV Adolescent PrEP and Prevention Study (CHAPS) is a mixed-methods research program investigating the acceptability and feasibility of implementing daily and on-demand PrEP among young people in SSA. It also aims to determine an on-demand dosing schedule for insertive sex. For this paper, we explored preferences for daily versus on-demand PrEP amongst adolescents as part of CHAPS.

**Methods:**

Purposive sampling was used to recruit participants from Soweto and Cape Town (South Africa), Wakiso district (Uganda) and Chitungwiza (Zimbabwe). At the time of the study in 2018/2019, Uganda had not rolled out PrEP to the general population; in Zimbabwe, PrEP for young people was only available at selected sites with one located within the study recruitment area. In South Africa, PrEP was made available to selected high-risk groups. We conducted 60 in-depth interviews and 24 group discussions amongst young people aged 13–24 without HIV in South Africa, Uganda, and Zimbabwe. All in-depth interviews and group discussions were audio-recorded, transcribed verbatim and translated to English. Data were analysed using framework analysis. The main themes were centered around preferences for daily and on-demand PrEP.

**Results:**

Reasons for on-demand preferences included stigma, pill fatigue, adherence and side effects. Reasons for daily PrEP preferences included factors related to sexual risk behaviour, continuous protection against incidents of unintentional exposure, and the increased efficacy of a daily dose. Participants at all sites preferring daily PrEP identified the same reasons, with more males than females citing inadvertent blood contact or perceived increased efficacy. Similarly, participants at all sites preferring on-demand PrEP gave the same reasons for their preferences for on-demand PrEP; the exception was South Africans who did not mention the hope of having fewer side effects by not taking daily PrEP. Additionally, more males than females cited intermittent sex as a reason for opting for on-demand PrEP.

**Conclusions:**

Our study is the first known to explore and describe youth preferences for daily versus on-demand PrEP. While the choice is clear-cut, the reasons cited in the different options provide invaluable insights into their decisions, and the actual and perceived facilitators and barriers to access to PrEP. Further education is needed amongst young people, not only about PrEP but also in other areas of comprehensive sexuality education. Exploring all options of HIV prevention is crucial to provide a tailored, one-size-does-not-fit-all approach to adolescent care in SSA to reduce and, the continued and increasing risk of this preventable infection.

## Introduction

Adolescents (10–19 years) and young people (10–29 years) continue to be disproportionately affected by HIV worldwide, with sub-Saharan Africa (SSA) remaining the most affected region [1]. HIV remains the fourth-leading cause of death in adolescents in African countries [2, 3]. In 2019, 2.8 million adolescents were living with HIV, with a further 320,000 becoming infected with the virus, amounting to 10% of all new infections globally [4]. Adolescents and young people in SSA remain vulnerable to a preventable chronic infection. Recent estimates by UNICEF indicate that nearly nine out of ten children and adolescents living with HIV are in SSA, with one in four new infections occurring in adolescent girls and young women [4].

An intervention for the prevention of HIV infection is pre-exposure prophylaxis (PrEP) [5], consisting of antiretroviral (ARV) drugs taken orally daily [6] for those at substantial risk of acquiring HIV. An on-demand regimen has also been shown to be effective in men who have sex with men (MSM) [7]. With significant evidence for efficacy in preventing HIV, ranging from 44%–86% [5, 8, 9], as well as its safety profile, PrEP is a valuable addition to current prevention strategies [10]. In SSA, several PrEP implementation research and demonstration projects are underway [11, 12]. However, despite recent progress, the availability and scale-up of PrEP remain sub-optimal, primarily owing to competition with other treatment programs for prioritization, resources and funding [13].

By 2020, 38 countries worldwide had approved the use of PrEP, including 10 African countries, with varying degrees of implementation in the public health sector [14]. Zimbabwe first introduced PrEP in May 2016 in the private sector and through demonstration projects for high-risk subpopulations, namely adolescent girls and young women, serodiscordant couples and MSM [15]. South Africa was the first country in the region to include PrEP in its national HIV plan, with roll-out commencing in June 2016 in a step-wise approach to key populations, starting with female sex workers, MSM, and then university students [16–21]. In May 2018, PrEP was offered to sexually active, HIV-negative female adolescents, aged 15–24 years, in Cape Town, South Africa [16, 22]. Uganda followed in November 2016, with PrEP only available through demonstration sites and facilities accredited to offer the service [23]. Although incidence rates in each SSA country are sufficient in at least one population group to qualify for PrEP, 31 African countries remain without regulatory approval for PrEP [20]. While SSA has both the highest prevalence of HIV and the largest population of adolescents and young people, the implementation of PrEP remains limited for this group at risk of HIV infection.

Recent research has shown that young people respond better to age-specific HIV services [24, 25]. Behavioral HIV preventative methods employed in SSA include family-based support groups, youth-led advocacy campaigns, and school engagement programmes, all of which show promising results [26–29]. The use of social protection methods, such as cash transfers, as a means to curb HIV incidence in adolescents have shown a reduction in intimate partner violence and HSV-2 transmission in South African adolescent girls [30, 31].

Despite cheap and effective HIV testing and prevention, an estimated 320 000 people were newly infected in Africa in 2021 [32]. PrEP has achieved tremendous impact in reducing HIV incidence in Europe and the America with less impact in Africa, where PrEP availability is more variable. Although on-demand PrEP for men is recommended by WHO [33], it is not included in African PrEP guidelines. The lack of PrEP data among men in SSA prevents policy and guideline change.

There is also pharmacokinetic modelling data to show that repeated doses are required to achieve what is thought to be adequate levels for protection in vaginal tissue (versus rectal tissue) [34]. For these reasons, on-demand PrEP is not recommended in people where HIV exposure may occur via vaginal mucosa [34]. In practice, young people may take intermittent doses due to the infrequency of their sexual exposure. Therefore, we explored participant preferences for daily and on-demand PrEP among male and female adolescents and youth in South Africa, Uganda and Zimbabwe.

## Materials and methods

The CHAPS study team used a qualitative methods research design by conducting in-depth interviews and group discussions.

### Study setting

At the time of the study in 2018/2019, Uganda had not rolled out PrEP to the general population; in Zimbabwe, PrEP for young people was only available at selected sites, with one located within the study recruitment area. In South Africa, PrEP was made available to selected high-risk groups.

### Sampling and recruitment

Purposive sampling was used to recruit participants from Soweto and Cape Town (South Africa), Wakiso district (Uganda) and Chitungwiza (Zimbabwe). South African and Zimbabwean participants were recruited from community groups, schools, churches, bars, taxi ranks and other public meeting places. In Uganda, participants were recruited at fish landing sites, with information on the study provided through local leaders, village health teams and project mobilizers.

Equal numbers of male and female volunteers aged 13–24 years were included, and all were required to have an HIV test. Those found to be living with HIV were provided with support and referral to care. Data were collected from those testing HIV negative, and included in this analysis.

### Ethical considerations

Study procedures were approved as per country requirements: Uganda–Uganda Virus Research Institute Research and Ethics Committee and Uganda National Council for Science and Technology; Zimbabwe–The Joint Research Ethics Committee for the University of Zimbabwe, College of Health Sciences and the Parirenyatwa Group of Hospitals, the Medical Research Council of Zimbabwe and the Research Council of Zimbabwe; South Africa–University of Cape Town Human Research Ethics Committee, and University of the Witwatersrand Human Research Ethics Committee.

Written informed consent was obtained from participants 18 years and older prior to study participation. Parental consent and participant assent were also required prior to any study activities for participants 18 years and younger, at the Johannesburg site only. Approval was obtained from ethics boards in Cape Town, Wakiso and Chitungwiza for a parental waiver of consent. Reimbursement was given to all participants for their time and participation according to the national requirements and guidelines of each site.

### Data collection procedures

Qualitative data were collected through 24 group discussions (eight per country), including a total of 189 participants, and 60 in-depth interviews (20 per country) between September 2018 and February 2019. Group discussions and interviews were conducted in-person at a confidential location at the respective research sites. The objectives and interview guides were the same for the group discussions and in-depth interviews. Data collection was stratified by age (13–17 and 18–24 years) and gender (male and female). In Cape Town, the group discussions included both males and females. All participants completed a socio-demographic questionnaire prior to participating in an interview or group discussion lasting approximately 40 minutes and 70 minutes, respectively.

Locally trained male and female social science interviewers, conversant in the local languages, first explained what PrEP is. Thereafter, interviewers used a semi-structured interview guide to elicit discussion about participants’ preferences for daily or on-demand PrEP. The following questions were asked: 1) How do you feel about taking medication every day to prevent HIV infections? 2) How would you feel about taking medication only when you need it to prevent HIV infection? 3) Would you prefer taking PrEP daily, if so why? 4) Would you prefer to take PrEP on-demand (before sex acts)? If so, why?

### Data analysis

All in-depth interviews and group discussions were audio-recorded, transcribed verbatim and translated to English. Trained and experienced qualitative researchers coded and analysed the data. Transcripts were coded in Excel for framework analysis [35]. Following the principles of the framework analysis approach, first, a researcher at each site read through four transcripts to achieve data familiarity. Thereafter, the PHRU team developed a codebook, which was shared with all sites for review, modification, development of code descriptions and finalization. After completion of the codebook, each site coded their site transcripts using a line-by-line technique to assign text to codes. A second researcher at each site also coded the same transcripts to identify codes using the line-by-line technique. Both researchers coding at each site then met to discuss the codes identified and discussed their similarities and differences and prepared their final codebook including the respective site codes.

Following this process, a researcher at the PHRU reviewed the Excel spreadsheet of coded data for all sites. The analysis process was focused on codes related to preferences for daily and on-demand PrEP. These codes were then categorized into themes and subthemes, which were shared with the study team for review in the Excel spreadsheet. Data analysis accounted for differences by site, gender, group discussion, and in-depth interview data.

## Results

### Participant characteristics

In total, 189 young people participated in the in-depth interviews and group discussions across all sites– 96 (50.7%) were female with a median age of 19 years, and 106 (56.6%) had a complete or incomplete high school education (data on level of education for Johannesburg, and Cape Town, South Africa and Zimbabwe only).

### PrEP preferences

The main themes were centered around preferences for daily and on-demand PrEP. We identified four subthemes related to preferences for daily PrEP: prevention against HIV infection due to unplanned consensual sex, perceived HIV prevention measures against sexual violence, inadvertent blood contact through accidents, and perceived increase in PrEP efficacy due to daily use. We also identified six subthemes related to preferences for on-demand PrEP use: fear of HIV-related stigma associated with daily pill use, perceived pill fatigue, non-adherence due to difficulty in taking a pill daily, forgetting daily pill doses, intermittent sex, and hope of fewer side effects by not taking PrEP daily. Where appropriate, data are presented by site and gender and we have ordered the findings according to the main themes and subthemes.

### Reasons for daily PrEP preference

#### HIV prevention

Across all sites, some participants from the group discussions and in-depth interviews gave the following reasons for choosing daily PrEP: prevention against HIV as a result of unplanned consensual sex, a preventative measure against HIV infection in cases of rape, and a precaution against HIV transmission following inadvertent blood contact. However, a few participants in Cape Town, Uganda and Zimbabwe indicated that their decision to use daily PrEP was to increase the drug’s effectiveness in the body, thus boosting their protection against HIV.

#### Prevention against HIV infection due to unplanned consensual sex

In Cape Town, Johannesburg and Zimbabwe, the group discussion (GD) and in-depth interview (IDI) participants’ preference for daily PrEP was driven by the perceived HIV risk among young people engaging in high-risk sexual behaviour, including transactional sex. In addition, both males and females said that sex is often not planned in advance. Females from Zimbabwe and Johannesburg explained why they would prefer to take PrEP daily:

*“As for myself I like taking [PreP] every day. Because of the issue of not knowing. Maybe, I might meet a guy who would want to give me money. So, it is better for me to take it beforehand so that when I have sex with him, I will not be infected by that sickness at all.” (**GD–females, 13–15 years, Zimbabwe**)*

*“The reason is because you don’t choose when you will have sex just like xxx mentioned that things such as Nandos, alcohol, drugs and alcohol, and the next thing you are kissing, leading to sex.” **(GD, females, 18–24 years, Soweto)***

One male participant aged 22 years in Uganda indicated that as a result of not knowing the HIV status of some of his sexual partners, daily PrEP would protect him from HIV infection.

#### Perceived HIV prevention measures against sexual violence

Group discussion and in-depth interview male and female participants across all sites expressed their fear of sexual violence, such as rape, which could result in HIV infection. These participants preferred to opt for daily PrEP to have a peace of mind for protection at all times. A female from Zimbabwe described how daily PrEP could be advantegous:

*“I want to take it [PrEP] every day because my friends, what they say about sex. So, they can give you a drink without yourself knowing what it contains. Maybe it has been laced with drugs and you end up having sex, so it’s wise to be always protected.” **(GD, males, 13–15 years, Zimbabwe**)*

A female participant from Cape Town described another situation in which she would prefer to take PrEP daily:

*“I would take it [PrEP] very frequently because I don’t know what might happen. Maybe, I could get out of here, take a cab to go somewhere and get raped and get HIV. Whereas if I had been eating it [PrEP] every day, maybe it would have helped me.” **(GD, mixed gender, 13–20 years, Cape Town)***

#### Inadvertent blood contact

Another motivation to opt for daily PrEP for some, mainly male, participants across all sites, raised primarily during the group discussions, was the fear of being involved in an accident. It included being involved in a motor vehicle accident and coming into contact with someone else’s blood, increasing the risk of HIV transmission. A male from Zimbabwe described a situation in which he or other young people could be in danger:

*“Myself on the issue of taking PrEP daily, people will like that because you encounter different things every day. Like you board a commuter omnibus and its involved in an accident. Knowing that you are on daily PrEP, so knowing that you are taking PrEP, you will be glad that you are on PrEP. Had you not been taking PrEP, you will be infected.” (**GD, males, 13–15 years, Zimbabwe**)*

One male participant from Zimbabwe mentioned the continuous protection daily PrEP provided for unforseen incidents, e.g., a needle prick:

*“Or you borrow a needle to sew your trousers and the needle pricks you. So knowing that you are taking PrEP you will be glad that you are on PrEP. Had you not been taking PrEP, you will be infected.” (**GD, males, 13–15 years, Zimbabwe**)*

#### Perceived increase in PrEP efficacy due to daily use

Most GD male participants across all sites stated that using PrEP daily would increase the pill’s effectiveness and enhance their level of HIV protection. They believed taking daily PrEP ensured they were well protected against HIV infection compared to on-demand PrEP. Males from Uganda and Zimbabwe explained as follows:

*“I say daily PrEP is better because your body can remain active and more protected when you are taking it [PrEP] than when you are taking on-demand PrEP. If you reach time for having sex, your body can be more protected even if you decide not to take it [PrEP].” **(GD, males, 13–17 years, Uganda)***

*“I think taking it [PrEP] every day is good because the on-demand pill might delay in getting into the system whilst you think you are protected. The everyday pill will therefore be effective since it’s already in the system.” **(GD, males, 16–18 years, Zimbabwe)***

### Reasons for on-demand PrEP preference

#### Fear of HIV-related stigma associated with daily pill use

Generally, male and female participants from the group discussions and in-depth interviews across all sites were interested in using some form of HIV prevention method. Some indicated that using daily PrEP would be difficult due to the fear of HIV-related stigma attached to taking a pill daily. Therefore, many participants preferred to opt for an on-demand PrEP regimen. There was consensus among participants that people may assume they are HIV positive and on ARVs if they took daily PrEP:

*“That daily PrEP will bring problems for us because you are equal to someone swallowing ARVs. I think the on-demand PrEP is the best option needed, and it’s the one I will use. But for the other one, you will be like an HIV positive person.” (**GD, females, 19–24 years, Uganda)***

*“And sometimes I am out partying, and I have to take the pills. Some people, they jump into conclusions there and there that xxx is positive.” **(GD, mixed gender,15–23 years, Cape Town)***

**Perceived pill fatigue.** Several female and male participants from Uganda, Soweto and Zimbabwe stated that on-demand PrEP would be the preferred option of HIV prevention as they would get tired of taking a pill daily:

*“Since you are not engaging sexually every day, you may end up seeing it as an unnecessary burden.” **(IDI, Male, 22 years, Zimbabwe)***

In general, participants from the group discussions and in-depth interviews admitted that they found it difficult to take pills, particularly on a daily basis. This was the challenge identified by some participants from Cape Town, Soweto and Zimbabwe:

*“I feel like using it [PrEP] only when you need it because taking a pill is hard when you don’t feel, you are not sick, right? So, you do not feel a headache or anything. Taking a pill is hard and it is forgettable, although it is said we must set an alarm and so on; it is hard. You become lazy.” **(IDI, female, 20 years, Cape Town)***

*“I will add to what was mentioned by xxx on taking every day, ah, it’s difficult I cannot do that. Pills are difficult to take every day. If PrEP is going to be like that, then I cannot take it [PrEP].” **(GD, females, 22–24 years, Zimbabwe)***

#### Forgetting to take a pill daily

Several group discussion female participants across all sites indicated they would find it hard to remember to take a pill daily. Therefore, they perceived on-demand PrEP as a more user-friendly and acceptable method of HIV prevention compared with daily PrEP. Females from Zimbabwe and Uganda described situations in which they would forget to take PrEP daily:

*“Personally, taking it [PrEP] every day for me is a struggle because sometimes I go to the bar and get drunk and I then won’t remember to take it [PrEP].” (**GD, females, 16–18 years, Zimbabwe)***

*“The daily PrEP needs someone who is not reluctant. Personally, I can’t try that one because I know sometimes I become so busy and I may forget. So, am kindly asking that they should give me on-demand PrEP rather than daily PrEP.” **(GD, females, 18–24 years, Uganda)***

#### Intermittent sex

Male in-depth interview participants reasoned that since they were not having sex frequently, they did not feel the need to take PrEP when not engaging in sexual activity. For these young people, taking on-demand PrEP would be more suitable. This was expressed mostly by male participants across all sites:

*“I will feel good about it [PrEP] because in a month, I have sex only once. That means I will need on-demand PrEP, not daily PrEP.” **(IDI, male, 17 years, Uganda)***

*“And I don’t see the reason why I should take it [PrEP] when I know I am not going to have sex; I am not going to be at risk.” **(IDI, male, 16 years, Cape Town)***

#### Hope of fewer side effects by not taking PrEP

A few male participants from Uganda and Zimbabwe were troubled by the potential increased risk of experiencing side effects and the fear that regular and daily use of PrEP might render the drug ineffective. Taking on-demand PrEP was thus perceived as reducing the level of drugs in one’s system, thereby reducing the side effects:

*“At times, issues to do with protection, we hear a lot of people in the community complaining that ever since they started taking pills, they have experienced side effects. That is one of the reasons why I prefer using them [PrEP] only on that day, not to take them daily. Using it [PrEP] for that day only will limit the amount of tablets in my body.” **(IDI, male, 21 years, Zimbabwe)***

*“Taking tablets daily, in most cases, reaches a point when they are no longer absorbable and crystallize and sometimes cause illnesses. And so, taking the tablets on demand makes absorption easier.” (**GD, males, 18–24 years, Uganda)***

Overall, we identified four subthemes and six subthemes related to preferences for daily or on-demand PrEP, respectively. Participants at all sites who preferred daily PrEP identified the same reasons, with more males than females citing inadvertent blood contact or perceived increased efficacy. Similarly, participants at all sites with a preference for on-demand PrEP gave the same reasons; an exception was the South Africans, who did not mention the hope of having fewer side effects by not taking daily PrEP. In addition, more males than females cited intermittent sex time as a reason for opting for on-demand PrEP.

## Discussion

Youth from our study preferred the on-demand PrEP option overall. They related this to reducing the stigma of being HIV positive, pill fatigue and forgetting to take PrEP daily, the hope for reduced side effects and the ability to tailor PrEP use to their sexual lifestyle. Participants preferring daily PrEP related its usefulness to frequent unplanned sex and peers engaging in risky sexual activities. In addition, they perceived daily doses of PrEP as protecting them when they did not know the HIV status of their romantic partners, lived in fear of sexual violence or wanted continuous protection against incidences of inadvertent blood contact. They also believed that, by taking a pill daily (as opposed to on-demand PrEP), they were increasing the overall effectiveness of PrEP.

PrEP has clearly been shown to be effective and feasible with approval and endorsement for daily and on-demand use, respectively, by WHO [32]. The SEARCH study has shown daily PrEP delivery to be feasible in SSA, as also evidenced by the roll-out of services in Zimbabwe, South Africa and Uganda [36]. Efficacy has been shown to be as high as 92% from blood detection of tenofovir in the iPrEx study that included MSM and transgender women with South Africa as one of six international study sites [9]. The Partner PrEP study conducted amongst HIV-serodiscordant couples in Kenya and Uganda showed that oral daily PrEP protected heterosexual males and females against HIV infection, while females showed better adherence towards PrEP use [5]. However, the VOICE study in women only, and based in South Africa, Uganda and Zimbabwe, failed to demonstrate the effectiveness of oral daily PrEP [37, 38]. This stark contrast was attributed to low adherence levels among participants in the VOICE study [39].

Qualitative studies showed that adherence was related to participants’ concerns about efficacy, the fear of being seen to be taking HIV drugs and the association with having HIV, and believing rumours within communities, including that PrEP was harmful, caused infertility or gave one HIV [40]. Furthermore, those most in need of PrEP were found least likely to adhere [41].

On-demand PrEP studies have included young people, but in two studies, only from 18 years upwards. A South African study evaluating daily oral PrEP versus two different on-demand PrEP regimens in women supported the recommendation of daily over on-demand PrEP [42], unlike the landmark IPERGAY trial showing the efficacy of the on-demand regimen in MSM [7]. The on-demand PrEP regimen provides efficacy comparable to daily dosing and in the highest risk areas of HIV transmission, i.e., anal sexual intercourse. Concerns cited within the studies were mainly around adherence. However, data is needed on adolescent preferences and understanding these choices to provide the entire HIV prevention cascade to a vulnerable group at increased risk of HIV infection in SSA. Our study fills this gap. Our study data provide contextual information on youth preferences to take PrEP. By focusing on youth, we plan to design a future trial of PrEP among this key population.

Demonstration and implementation projects in Africa, albeit only in adolescent girls and young women, reveal the need for strategies for adherence and persistence of PrEP [43]. Long-acting interventions, e.g., injections, rings and implants, are being studied for HIV prevention with encouraging results. Adolescents are particularly enthusiastic about having an alternative to pills [44]. However, history has shown that the implementation of novel strategies often lags significantly compared with higher-income countries. For example, the contraceptive implant was introduced in the UK in 1993 and in the public sector in South Africa almost 20 years later [45], while the antiretroviral dolutegravir was rolled out in Uganda five years after it was first used in the USA [23, 46]. Exploring an alternative regimen for oral PrEP, which is already known to be effective and safe in high-risk groups and adolescents as a daily tablet, can provide additional choices for adolescents’ unique, different and complex needs.

When considering daily versus on-demand PrEP, their efficacy and safety has been found to be similar, albeit that the on-demand regimen has only been studied in gay or bisexual men. The other potential advantages and disadvantages of on-demand PrEP lie in subjective preferences, mainly from studies of MSM, who cite ease of administration (number, duration, timing) and predictability of sexual activity [47–50]. Further studies have shown the perceived advantages of PrEP, regardless of the regimen, as a tool for empowerment, the ability to take control of one’s sexual health risk within a relationship and also with multiple partners [51]. It has also been reported as a risk reduction strategy when needle exchange for drug users was not readily available [52].

On demand oral PrEP has not been evaluated in men in Africa [33]; this is despite men from SSA preferring these prevention choices over daily tablets if available [53]. To date, HIV prevention efforts in sub-Saharan Africa (SSA) have been female focused, leaving men the largest unaddressed gap in HIV services [54]. Men fare worse than women in uptake of HIV testing and ART initiation[55], as well as engagement and retention in HIV prevention/treatment programmes [56–58]. As such, HIV incidence in men has not fallen and the WHO/UNAIDS has launched the Global Men and HIV Technical Working Group (MENHT) [59].

Finally, in adolescents and youth specifically, stigma and discrimination remain a concern in light of misconceptions about the association of PrEP use with being “promiscuous” and having HIV, given that the drug combination for PrEP is also used to treat HIV [60]. Disclosure to parents and/or partners of taking PrEP was also described as a disadvantage, albeit by healthcare workers in relation to adolescents and youth [61].

### Study limitations

As PrEP had already been rolled out in the recruitment areas, real and perceived information about PrEP was already in circulation. We based the findings of our study on perceived rather than actual behaviours, not only of the individual participants but also of their peers. The mixing of genders for the Cape Town data collection may have created response bias as participants may have exaggerated or under-reported in their responses. Data on level of education were not collected for participants from Uganda. Additionally, participants were not asked about their sexual activity, pregnancy history and prior or current PrEP use, which may have influenced their responses regarding their PrEP preferences.

## Conclusion

Our study was one of the first to explore and describe youth preferences for daily versus on-demand PrEP in sub-Saharan Africa. In particular, this study provides insights on PrEP from male adolescents to narrow the gap of including this population in the HIV prevention efforts. The reasons cited for the different PrEP options provide invaluable insights into their decisions, and the actual and perceived facilitators and barriers to access to PrEP. It further highlights current strategies are not fully addressing the needs for optimal PrEP implementation. The voice of adolescents as per our findings should be used in conjunction with healthcare providers and other key stakeholders to ensure needs are met through education among young people in healthcare and other settings, while complimenting this with ensuring providers are equipped with the necessary training and resources. A holistic approach, with combined prevention approaches that includes other areas of comprehensive sexuality education is ultimately needed to prevent HIV as well as other risks within the sexual health of young people. Exploring all options for HIV prevention is crucial to provide a tailored, one-size-does-not-fit-all approach to adolescent care in SSA and to reduce and, ultimately, eliminate the continued and increasing risk of this preventable infection.

## References

[1] UNAIDS. Active involvement of young people is key to ending the AIDS epidemic by 2030. 2015 [cited 2020 Dec 15]. Available from: https://www.unaids.org/en/resources/presscentre/featurestories/2015/august/20150812_PACT#:~:text=Update-,Active%20involvement%20of%20young%20people%20is%20key,the%20AIDS%20epidemic%20by%202030&text=to%20lifesaving%20treatm-,While%20major%20advances%20have%20been%20made%20in%20responding%20to%20HIV,needs%20to%20be%20scaled%20up.

[2] WHO. Global Health Estimates 2016: Deaths by Cause, Age, Sex, by Country and by Region, 2000–2016. Geneva, Switzerland: WHO, 2018. Available from: https://www.who.int/healthinfo/global_burden_disease/estimates/en/.

[3] Armstrong A, Nagata JM, Vicari M, Irvine C, Cluver L, Sohn AH, et al. A Global Research Agenda for Adolescents Living With HIV. J Acquir Immune Defic Syndr. 2018;78(1):16–21. doi: 10.1097/qai.0000000000001744 29994915 PMC6075888

[4] UNICEF. 2020 World AIDS Day Report. New York, USA: UNICEF, 2020. Available from: http://www.childrenandaids.org/sites/default/files/2020-12/2020%20World%20AIDS%20Day%20Report.pdf.

[5] Baeten JM, Donnell D, Ndase P, Mugo NR, Campbell JD, Wangisi J, et al. Antiretroviral prophylaxis for HIV prevention in heterosexual men and women. N Engl J Med. 2012;367(5):399–410. doi: 10.1056/NEJMoa1108524 22784037 PMC3770474

[6] McCormack S, Dunn DT, Desai M, Dolling DI, Gafos M, Gilson R, et al. Pre-exposure prophylaxis to prevent the acquisition of HIV-1 infection (PROUD): effectiveness results from the pilot phase of a pragmatic open-label randomised trial. Lancet. 2016;387(10013):53–60. doi: 10.1016/S0140-6736(15)00056-2 26364263 PMC4700047

[7] Molina JM, Capitant C, Spire B, Pialoux G, Cotte L, Charreau I, et al. On-Demand Preexposure Prophylaxis in Men at High Risk for HIV-1 Infection. N Engl J Med. 2015;373(23):2237–46. doi: 10.1056/NEJMoa1506273 26624850

[8] Choopanya K, Martin M, Suntharasamai P, Sangkum U, Mock PA, Leethochawalit M, et al. Antiretroviral prophylaxis for HIV infection in injecting drug users in Bangkok, Thailand (the Bangkok Tenofovir Study): a randomised, double-blind, placebo-controlled phase 3 trial. Lancet. 2013;381(9883):2083–90. doi: 10.1016/S0140-6736(13)61127-7 23769234

[9] Grant RM, Lama JR, Anderson PL, McMahan V, Liu AY, Vargas L, et al. Preexposure chemoprophylaxis for HIV prevention in men who have sex with men. N Engl J Med. 2010;363(27):2587–99. doi: 10.1056/NEJMoa1011205 21091279 PMC3079639

[10] Pilkington V, Hill A, Hughes S, Nwokolo N, Pozniak A. How safe is TDF/FTC as PrEP? A systematic review and meta-analysis of the risk of adverse events in 13 randomised trials of PrEP. J Virus Erad. 2018;4(4):215–24. doi: 10.1056/NEJMoa1108524 30515300 PMC6248833

[11] Cowan FM, Delany-Moretlwe S, Sanders EJ, Mugo NR, Guedou FA, Alary M, et al. PrEP implementation research in Africa: what is new? J Int AIDS Soc. 2016;19(7):21101. doi: 10.7448/ias.19.7.2110121101 27760680 PMC5071780

[12] AVAC. Ongoing and planned PrEP demonstration and implementation studies. 2019 [cited 2021 May 28]. Available from: https://www.avac.org/resource/ongoing-and-planned-prep-demonstration-and-implementation-studies.

[13] Ahmed N, Pike C, Bekker LG. Scaling up pre-exposure prophylaxis in sub-Saharan Africa. Curr Opin Infect Dis. 2019;32(1):24–30. doi: 10.1097/QCO.0000000000000511 30461452

[14] PrEPWatch. About PrEP. 2020 [cited 2020 Dec 15]. Available from: https://www.prepwatch.org/about-prep/.

[15] The National Medicine and Therapeutics Policy Advisory Committee TAaTD, Ministry of Health and Child Care. Guidelines for Antiretroviral Therapy for the Prevention and Treatment of HIV in Zimbabwe. Harare, Zimbabwe: Ministry of Health and Child Care, 2016. Available from: https://depts.washington.edu/edgh/zw/vl/project-resources/ZIM_ART_Guidelines_2016_-_review_final.pdf.

[16] South African National AIDS Council. National Strategic Plan on HIV, STIs and TB 2012–2016. Pretoria, South Africa: SANAC, 2012. Available from: https://www.gov.za/sites/default/files/gcis_document/201409/national-strategic-plan-hiv-stis-and-tb0.pdf.

[17] SANAC. South Africa National Sex Worker HIV Plan (2016–2019). South Africa: SANAC, 2016. Available from: https://www.prepwatch.org/resource/sa-sex-worker-hiv-plan/.

[18] SANAC. Monitoring and evaluation plan for the national strategic plan on HIV, TB and STI (2017–2022). Pretoria, South Africa: SANAC, 2017. Available from: https://sanac.org.za/the-national-strategic-plan/.

[19] PrEPWatch. South Africa. 2021 [cited 2021 Apr 14]. Available from: https://www.prepwatch.org/country/south-africa/.

[20] PrEPWatch. The Global PrEP Tracker. 2020 [cited 2021 Apr 14]. Available from: https://data.prepwatch.org.

[21] Department of Health Republic of South Africa. National Policy on HIV Pre-exposure Prophylaxis (PrEP) and Test and Treat (T&T). Pretoria, South Africa: DoH, 2016. Available from: https://sahivsoc.org/Files/PREP%20and%20TT%20Policy%20-%20Final%20Draft%20-%205%20May%202016%20(HIV%20news).pdf.

[22] Bekker L-G, Brown B, Joseph-Davey D, Gill K, Moorhouse M, Delany-Moretlwe S, et al. Southern African guidelines on the safe, easy and effective use of pre-exposure prophylaxis: 2020. Southern African journal of HIV medicine. 2020;21(1):1152–. doi: 10.4102/sajhivmed.v21i1.1152 33354364 PMC7736681

[23] Ministry of Health Uganda. Consolidated guidelines for the prevention and treatment of HIV and AIDS in Uganda. Kampala, Uganda: Government of Uganda, 2018. Available from: https://uac.go.ug/sites/default/files/Consolidated%20HIV%20Guidelines%202020.pdf.

[24] Asia Pacific Network of People Living with HIV/AIDS. Lost in Transitions: Current issues faced by adolescents living with HIV in Asia Pacific. Bangkok: APN+, 2013. Available from: https://www.aidsdatahub.org/sites/default/files/resource/lost-transitions-current-issues-adolescents-hiv-asia.pdf.

[25] WHO. Global Accelerated Action for the Health of Adolescents (AA-HA!): guidance to support country implementation. Geneva, Switzerland: World Health Organization, 2017. Available from: https://apps.who.int/iris/bitstream/handle/10665/255415/9789241512343-eng.pdf;jsessionid=8F21868B727CA864C5100E66082EBFCF?sequence=1.

[26] Bhana A, Mellins CA, Petersen I, Alicea S, Myeza N, Holst H, et al. The VUKA family program: piloting a family-based psychosocial intervention to promote health and mental health among HIV infected early adolescents in South Africa. AIDS Care. 2014;26(1):1–11. doi: 10.1080/09540121.2013.806770 23767772 PMC3838445

[27] Kogan SM, Yu T, Brody GH, Chen YF, DiClemente RJ, Wingood GM, et al. Integrating condom skills into family-centered prevention: efficacy of the Strong African American Families-Teen program. J Adolesc Health. 2012;51(2):164–70. doi: 10.1016/j.jadohealth.2011.11.022 22824447 PMC3404410

[28] Denison JA, Pettifor A, Mofenson LM, Kasedde S, Marcus R, Konayuma KJ, et al. Youth engagement in developing an implementation science research agenda on adolescent HIV testing and care linkages in sub-Saharan Africa. Aids. 2017;31(3):195–201. doi: 10.1097/QAD.0000000000001509 28665877 PMC5497774

[29] Campbell C, Andersen L, Mutsikiwa A, Madanhire C, Nyamukapa C, Gregson S. Can Schools Support HIV/AIDS-Affected Children? Exploring the ’Ethic of Care’ amongst Rural Zimbabwean Teachers. PLoS One. 2016;11(1):e0146322. doi: 10.1371/journal.pone.0146322 26790103 PMC4720431

[30] Pettifor A, MacPhail C, Hughes JP, Selin A, Wang J, Gómez-Olivé FX, et al. The effect of a conditional cash transfer on HIV incidence in young women in rural South Africa (HPTN 068): a phase 3, randomised controlled trial. Lancet Glob Health. 2016;4(12):e978–e88. doi: 10.1016/S2214-109X(16)30253-4 27815148 PMC5626439

[31] Humphries H, Kharsany AB, Leask K, Ntombela F, Karim QA. The impact of conditional cash transfers in reducing HIV in adolescent girls and boys (RHIVA): the CAPRISA 007 matched pair, cluster randomised controlled trial. The CAPRISA Clinical Trials: HIV Treatment and Prevention: Springer; 2017. p. 77–89.

[32] UNAIDS. Global HIV & AIDS statistics—Fact sheet. 2021 [cited 2022 16 December]. Available from: https://www.unaids.org/en/resources/fact-sheet.

[33] World Health Organization. Guidelines on long-acting injectable cabotegravir for HIV prevention. Geneva, Switzerland: World Health Organization, 2022. Available from: https://www.who.int/publications/i/item/9789240054097. 36417549

[34] Bailey JL, Molino ST, Vega AD, Badowski M. A Review of HIV Pre-Exposure Prophylaxis: The Female Perspective. Infect Dis Ther. 2017;6(3):363–82. doi: 10.1007/s40121-017-0159-9 28600755 PMC5595773

[35] Gale NK, Heath G, Cameron E, Rashid S, Redwood S. Using the framework method for the analysis of qualitative data in multi-disciplinary health research. BMC Med Res Methodol. 2013;13:117. doi: 10.1186/1471-2288-13-117 24047204 PMC3848812

[36] Celum C, Baeten JM. Lessons on PrEP from the SEARCH study in east Africa. Lancet HIV. 2020;7(4):e219–e20. doi: 10.1016/S2352-3018(20)30003-5 32087151

[37] Baeten JM, Haberer JE, Liu AY, Sista N. Preexposure prophylaxis for HIV prevention: where have we been and where are we going? J Acquir Immune Defic Syndr. 2013;63(2):S122–9. doi: 10.1097/QAI.0b013e3182986f69 23764623 PMC3710117

[38] Murnane PM, Celum C, Mugo N, Campbell JD, Donnell D, Bukusi E, et al. Efficacy of preexposure prophylaxis for HIV-1 prevention among high-risk heterosexuals: subgroup analyses from a randomized trial. Aids. 2013;27(13):2155–60. doi: 10.1097/QAD.0b013e3283629037 24384592 PMC3882910

[39] Marrazzo JM, Ramjee G, Richardson BA, Gomez K, Mgodi N, Nair G, et al. Tenofovir-based preexposure prophylaxis for HIV infection among African women. N Engl J Med. 2015;372(6):509–18. doi: 10.1056/NEJMoa1402269 25651245 PMC4341965

[40] Van der Elst EM, Mbogua J, Operario D, Mutua G, Kuo C, Mugo P, et al. High acceptability of HIV pre-exposure prophylaxis but challenges in adherence and use: qualitative insights from a phase I trial of intermittent and daily PrEP in at-risk populations in Kenya. AIDS Behav. 2013;17(6):2162–72. doi: 10.1007/s10461-012-0317-8 23080358 PMC3690654

[41] Haberer JE. Current concepts for PrEP adherence in the PrEP revolution: from clinical trials to routine practice. Curr Opin HIV AIDS. 2016;11(1):10–7. doi: 10.1097/COH.0000000000000220 26633638 PMC4801217

[42] Bekker LG, Roux S, Sebastien E, Yola N, Amico KR, Hughes JP, et al. Daily and non-daily pre-exposure prophylaxis in African women (HPTN 067/ADAPT Cape Town Trial): a randomised, open-label, phase 2 trial. Lancet HIV. 2018;5(2):e68–e78. doi: 10.1016/S2352-3018(17)30156-X 28986029 PMC6107917

[43] Celum CL, Delany-Moretlwe S, Baeten JM, van der Straten A, Hosek S, Bukusi EA, et al. HIV pre-exposure prophylaxis for adolescent girls and young women in Africa: from efficacy trials to delivery. J Int AIDS Soc. 2019;22 (4):e25298. doi: 10.1002/jia2.25298 31328444 PMC6643076

[44] Gill K, Happel AU, Pidwell T, Mendelsohn A, Duyver M, Johnson L, et al. An open-label, randomized crossover study to evaluate the acceptability and preference for contraceptive options in female adolescents, 15 to 19 years of age in Cape Town, as a proxy for HIV prevention methods (UChoose). J Int AIDS Soc. 2020;23(10):e25626. doi: 10.1002/jia2.25626 33034421 PMC7545920

[45] FPA. Contraception: past, present and future. London, United Kingdom: FPA, 2010. Available from: https://www.fpa.org.uk/factsheets/contraception-past-present-future#:~:text=Implants,was%20introduced%20in%20the%20UK.

[46] Mullick S, Chersich MF, Pillay Y, Rees H. Introduction of the contraceptive implant in South Africa: Successes, challenges and the way forward. S Afr Med J. 2017;107(10):812–4. doi: 10.7196/samj.2017.v107i10.12849 29397679

[47] Noret M, Balavoine S, Pintado C, Siguier M, Brun A, Bauer R, et al. Daily or on-demand oral tenofovir disoproxil fumarate/emtricitabine for HIV pre-exposure prophylaxis: experience from a hospital-based clinic in France. Aids. 2018;32(15):2161–9. doi: 10.1097/QAD.0000000000001939 30212403

[48] Macapagal K, Nery-Hurwit M, Matson M, Crosby S, Greene GJ. Perspectives on and preferences for on-demand and long-acting PrEP among sexual and gender minority adolescents assigned male at birth. Sex Res Social Policy. 2021;18(1):39–53. doi: 10.1007/s13178-020-00441-1 33456624 PMC7810244

[49] Kwan TH, Lui GCY, Lam TTN, Lee KCK, Wong NS, Chan DPC, et al. Comparison between daily and on-demand PrEP (pre-exposure prophylaxis) regimen in covering condomless anal intercourse for men who have sex with men in Hong Kong: A randomized, controlled, open-label, crossover trial. J Int AIDS Soc. 2021;24(9):e25795. doi: 10.1002/jia2.25795 34473402 PMC8412015

[50] Tan DH-S. PrEP on demand or every day? The Lancet HIV. 2017;4(9):e379–e80. doi: 10.1016/S2352-3018(17)30088-7 28747273

[51] Bond KT, Gunn AJ. Perceived Advantages and Disadvantages of Using Pre-Exposure Prophylaxis (PrEP) among Sexually Active Black Women: An Exploratory Study. J Black Sex Relatsh. 2016;3(1):1–24. doi: 10.1353/bsr.2016.0019 28725660 PMC5512598

[52] Arkell C. Oral pre-exposure prophylaxis (PrEP) fact sheet. 2016 [cited 2022 Feb 23]. Available from: https://www.thebodypro.com/article/oral-pre-exposure-prophylaxis-prep-fact-sheet.

[53] Dietrich JJ, Atujuna M, Tshabalala G, Hornschuh S, Mulaudzi M, Koh M, et al. A qualitative study to identify critical attributes and attribute-levels for a discrete choice experiment on oral pre-exposure prophylaxis (PrEP) delivery among young people in Cape Town and Johannesburg, South Africa. BMC Health Services Research. 2021;21(1):17. doi: 10.1186/s12913-020-05942-8 33407395 PMC7788832

[54] Giguère K, Eaton JW, Marsh K, Johnson LF, Johnson CC, Ehui E, et al. Trends in knowledge of HIV status and efficiency of HIV testing services in sub-Saharan Africa, 2000–20: a modelling study using survey and HIV testing programme data. Lancet HIV. 2021;8(5):e284–e93. doi: 10.1016/S2352-3018(20)30315-5 33667411 PMC8097636

[55] Cornell M, Majola M, Johnson LF, Dubula-Majola V. HIV services in sub-Saharan Africa: the greatest gap is men. Lancet. 2021;397(10290):2130–2. doi: 10.1016/S0140-6736(21)01163-6 34087108

[56] Laurent C, Dembélé Keita B, Yaya I, Le Guicher G, Sagaon-Teyssier L, Agboyibor MK, et al. HIV pre-exposure prophylaxis for men who have sex with men in west Africa: a multicountry demonstration study. Lancet HIV. 2021;8(7):e420–e8. doi: 10.1016/S2352-3018(21)00005-9 34048794

[57] Zhang J, Li C, Xu J, Hu Z, Rutstein SE, Tucker JD, et al. Discontinuation, suboptimal adherence, and reinitiation of oral HIV pre-exposure prophylaxis: a global systematic review and meta-analysis. Lancet HIV. 2022;9(4):e254–e68. doi: 10.1016/S2352-3018(22)00030-3 35364026 PMC9124596

[58] Phiri MM, Schaap A, Simwinga M, Hensen B, Floyd S, Mulubwa C, et al. Closing the gap: did delivery approaches complementary to home-based testing reach men with HIV testing services during and after the HPTN 071 (PopART) trial in Zambia? J Int AIDS Soc. 2022;25(1):e25855. doi: 10.1002/jia2.25855 35001530 PMC8743361

[59] Phillips AN, Cambiano V, Johnson L, Nakagawa F, Homan R, Meyer-Rath G, et al. Potential Impact and Cost-Effectiveness of Condomless-Sex-Concentrated PrEP in KwaZulu-Natal Accounting for Drug Resistance. J Infect Dis. 2021;223(8):1345–55. doi: 10.1093/infdis/jiz667 31851759 PMC8064039

[60] Rousseau E, Katz AWK, O’Rourke S, Bekker LG, Delany-Moretlwe S, Bukusi E, et al. Adolescent girls and young women’s PrEP-user journey during an implementation science study in South Africa and Kenya. PLoS One. 2021;16(10):e0258542. doi: 10.1371/journal.pone.0258542 34648589 PMC8516266

[61] Lanham M, Ridgeway K, Mireku M, Nhamo D, Pillay D, Murire M, et al. Health care providers’ attitudes toward and experiences delivering oral PrEP to adolescent girls and young women in Kenya, South Africa, and Zimbabwe. BMC Health Serv Res. 2021;21(1):1112. doi: 10.1186/s12913-021-06978-0 34663320 PMC8522219

